# Use of WhatsApp®, for distance teaching during COVID-19 pandemic: Experience and perception from a sub-Saharan African setting

**DOI:** 10.1186/s12909-021-02953-9

**Published:** 2021-10-02

**Authors:** Dominique Enyama, Eric Vounsia Balti, Sylvain Raoul Simeni Njonnou, Christian Ngongang Ouankou, Fernando Kemta Lekpa, Diomede Noukeu Njinkui, Jovanny Tsuala Fouogue, Jeanne Mayouego Kouam, Guy Sedar Singor Njateng, Bruno Kenfack, Pierre Watcho, Simeon Pierre Choukem

**Affiliations:** 1 Faculty of Medicine and Pharmaceutical Sciences, University of Dschang, Dschang, Cameroon; 2 Department of Paediatrics, Douala Gynaeco-Obstetric and Paediatric Hospital, Douala, Cameroon; 3 The University of Dschang Taskforce for the Elimination of COVID, Dschang, Cameroon; 4 Department of Internal Medicine, Universiteit Ziekenhuis Brussel, Vrije Universiteit Brussel, Brussels, Belgium; 5 Yaoundé University Teaching Hospital, Yaoundé, Cameroon; 6 Department of Internal Medicine, Douala General Hospital, Douala, Cameroon; 7 Department of Obstetrics and Gynaecology, Bafoussam Regional Hospital, Bafoussam, Cameroon; 8 Department of Biochemistry, Faculty of Sciences, University of Dschang, Dschang, Cameroon; 9 Health and Human Development (2HD) Research Network, Douala, Cameroon

**Keywords:** Distance education, graduate education, mobile applications, online-learning, perception, social media

## Abstract

**Background:**

In the midst of the COVID-19 pandemic, to palliate to the lockdown and cover academic programs, the faculty of medicine and pharmaceutical sciences (FMPS) of the university of Dschang (UDs) in Cameroon has implemented e-learning using WhatsApp**®.**

**Aim:**

Describe the opinion of students and lecturers after its implementation of e-learning at the FMPS of UDs.

**Methods:**

We designed a uniform teaching scheme using WhatsApp**®** during the university lockdown. Students and members of the teaching staff of the FMPS of UDs were enrolled after receiving clear information on the study implementation. At the end of the online-teaching period of two and a half months, we surveyed our students and teaching staff. Sociodemographic characteristics and opinions about e-learning were collected using a standard questionnaire.

**Results:**

We enrolled 229 students and 40 lecturers of the FMPS. Students reported a decremented quality of internet connection (*p* < 0.001, p-homogeneity < 0.001) despite an increased expenditure related to internet use. Electronic devices were broadly used before the implementation of mobile learning. The use of course materials was significantly more challenging among students because of the size/format of lecture notes and internet connection/cost (all *p* < 0.05). Perception of discipline compared to classroom-based lessons was not significantly different among students compared to lecturers (all *p* > 0.05). While lecturers were mainly more comfortable conveying the contents of their lectures, students tended to be less prone to actively participate. The motivation and satisfaction of the latter group toward e-learning were modest compared to classroom-based lectures while their feedback about the organization was positive.

**Conclusions:**

E-learning using WhatsApp**®** could be an effective alternative to conventional classroom-based lessons in the context of COVID-19 pandemic. The use of a blended-learning program including classroom-based sessions could help improve its limitations.

**Supplementary Information:**

The online version contains supplementary material available at 10.1186/s12909-021-02953-9.

## Background

The novel Coronavirus disease (COVID-19) outbreak has led to a lockdown in almost all countries of the world. By March 18, 2020, 107 countries had implemented national school closure as a major preventive strategy [1]. In Cameroon, Central Africa, the government response strategy to the coronavirus pandemic included the closure of all public and private training institutions of the various levels of education, from nursery school to higher education, including vocational training centers and professional schools [2].

In this context, the faculty of medicine and pharmaceutical sciences (FMPS) of the university of Dschang (UDs) had to set up a strategy to deliver academic programs and to ensure the continuity of training. To comply with the need of social distancing during the COVID-19 pandemic, internet-based teaching was the most convenient strategy. Given the fact that the faculty was not prepared to face this challenge and considering the urgent need to find a solution, the choice felt on WhatsApp® that would be used as a teaching channel.

This social media offered the advantage of being widely used by both teachers and students before containment measures. It also offered a minimum of functionalities in terms of transferring text, video, sound and image files to users within classes organized in study groups. Finally, it offered the opportunity to be used both on smartphones and computers without the need of heavy logistics such as servers [3–5]. Moreover, it has been shown in another context to be more applicable in settings where there are challenges to obtain permanent or regular optimal internet bandwidth or speed [6].

The rapid implementation of this teaching strategy enabled to carry on with academic programs using e-learning as soon as from March 18, 2020, the day Cameroonian universities closed. The purpose of this study was to describe how e-learning was put in place and the perception as well as difficulties encountered both by students and faculty members of the FMPS during COVID-19 pandemic.

## Methods

### Study setting

A cross-sectional study was conducted among students and lecturers of the FMPS, from April 26 to May 4, 2020. This faculty is part of the UDs located in the city of Dschang, in the West Region of Cameroon since 2017. The UDs is one of the main universities in Cameroon with more than 600 lecturers and almost 30, 000 students. The population included in this study was recruited among students and lecturers (permanent and non-tenure track faculty members) of the FMPS. The study was approved by the institutional board of the UDs and the FMPS.

### Study population

Students and lecturers of the FMPS were approached and consecutively included regardless of study type and level for students and independently of administrative position or teaching subject for the lecturers. By the time of conduct of the study, the FMPS had 40 lecturers and 698 students. Clear information was given to all students and faculty members about the study purpose, adequate filling of questionnaires and participation modalities. All participants who responded to the questionnaire were included. Informed consent was obtained from all study participants and the study protocol was implemented according to the recommandations of the latest revision of the Helsinki declaration.

### Description of the WhatsApp® e-learning scheme

The implementation of e-learning was done using the same lectures’ schedule prior to the lockdown. No recommendation was given to the lecturers on how to conduct the lessons however, we did not expect any change in contain compared to in person lessons. Virtual classes pertaining to the various study levels were created through WhatsApp® groups. Those platforms were used by all lecturers who were invited to join the class at the day and time of the planned lecture. Lecture notes were first sent in Portable Document Format (PDF), word or powerpoint format and lessons were given via voice-mails. Interactions (questions and answers) during the lesson used the same channel or short message service as an alternative communication medium. The lessons were complemented by an electronic platform where all course materials were provided to students for further reading.

### Procedure

Data were collected using an anonymous online survey administered in both official languages (French and English) using Google Forms (Alphabet Inc., California, USA). Data complied with the terms and conditions of Google Forms. In an initial step, we conducted a pilot survey to optimize data collection forms. Responses to the pilot survey were excluded. All issues raised during that preliminary phase of the study were addressed. We then distributed the survey link to students directly via the various class representatives and lecturers via a WhatsApp® (Facebook Inc., California, USA) group. The class representatives disseminated the survey links in the respective official students’ WhatsApp® groups. WhatsApp® was already the primary mean of formal communication among students and lecturers of the FMPS. We collected the total number of students in each group to calculate the participation rate, link dissemination was confirmed by screenshots, and reminder messages were sent every week in the class groups to ensure a reasonable participation rate.

### Variables

Using standard questionnaires, data were collected on sociodemographic (age, gender study level) characteristics, challenges and the opinion of the study population about internet-based distance learning. Survey questions were a mix of open, close, and multiple-choice questions.

### Statistical analysis

The data collected were processed on Excel 2016 (Microsoft Corp., Washington, USA). We analyzed data using Stata 16 (StataCorp, Texas, USA). Figures were generated with GraphPad Prism 8 (San Diego, California, USA). Quantitative data are presented as median (interquartile range [IQR]) and qualitative data as frequencies and proportions. Group comparisons were performed with Chi-square or Fisher’s exact tests. Variations of the flow of internet access, expenditure and connection time for academic purposes after implementation of internet-based lectures were assessed using Bowker’s test for table symmetry. Statistical significance was adjudicated using Stuart-Maxwell test for marginal homogeneity. The threshold of significance was set at 0.05.

## Results

### Study population and internet access before online-based lectures

The study population included 229 students and 40 faculty members with median age (IQR): 20 (19–22) and 39 (36–43) years, respectively. This yielded a participation rate of 32.8 % and 100 % respectively among students and faculty members. Female students were more represented than their male counterparts, *n* = 140 (M/F ratio 0.64) while lecturers were more men than women, *n* = 29 (M/F ratio 2.6). The large majority of students were registered for biomedical sciences [n(%) = 100 (43.7)] followed by medical studies [(n%) = 84(36.7)]. Students in their first and second years of studies were more represented [n(%) = 77(33.62) and n(%) = 82 (35.8), respectively]. All students had internet access before the implementation of distance learning. The same was true for lecturers who had overall better internet access than students (overall *p* < 0.001), Table 1. While students reported significantly reduced quality of internet connection during the implementation of online courses than before (*p* < 0.001, p-homogeneity < 0.001), internet access was similar among faculty members before and during e-teaching (*p* = 0.549, p-homogeneity = 0.351).

**Table.1:** Characteristics of the study population

Characteristics	Students (*n* = 229)	Lecturers (*n* = 40)
Age, years	20 (19–22)	39 (36–43)
Gender, M/F (ratio)	89 (0.64)	29 (2.6)
Study path, n (%)		
Master studies*	22 (9.61)	-
Medical studies	84 (36.68)	-
Pharmacy studies	22 (10.04)	-
Biomedical sciences	100 (43.67)	-
Study level, n (%)		
First year	77 (33.62)	-
Second year	48 (20.96)	-
Third year	82 (35.81)	-
Fourth and fifth year	22 (9.61)	-
Average time on WhatsApp		
less than 2 h/day	14 (6.11)	9 (22.5)
2 to 4 h/day	47 (20.52)	14 (35)
4 to 6 h/day	68 (29.69)	9 (22.5)
6 to 8 h/day	33 (14.41)	3 (7.5)
8 to 10 h/day	20 (8.73)	2 (5.0)
10 to 12 h/day	24 (10.48)	2 (5.0)
More than 12 h/day	23 (10.04)	1 (2.5)
Internet access **		
Poor, n (%)	13 (5.68)	0 (0.00)
Average, n (%)	113 (49.34)	8 (20.00)
Good, n (%)	90 (39.30)	25 (62.50)
Very good, n (%)	13 (5.68)	7 (17.50)

Age is expressed as median (interquartile range), *clinical biology and public health, **overall *p* < 0.001.

### Devices used for internet access, financial and time burden of e-lectures

**Fig. 1:**
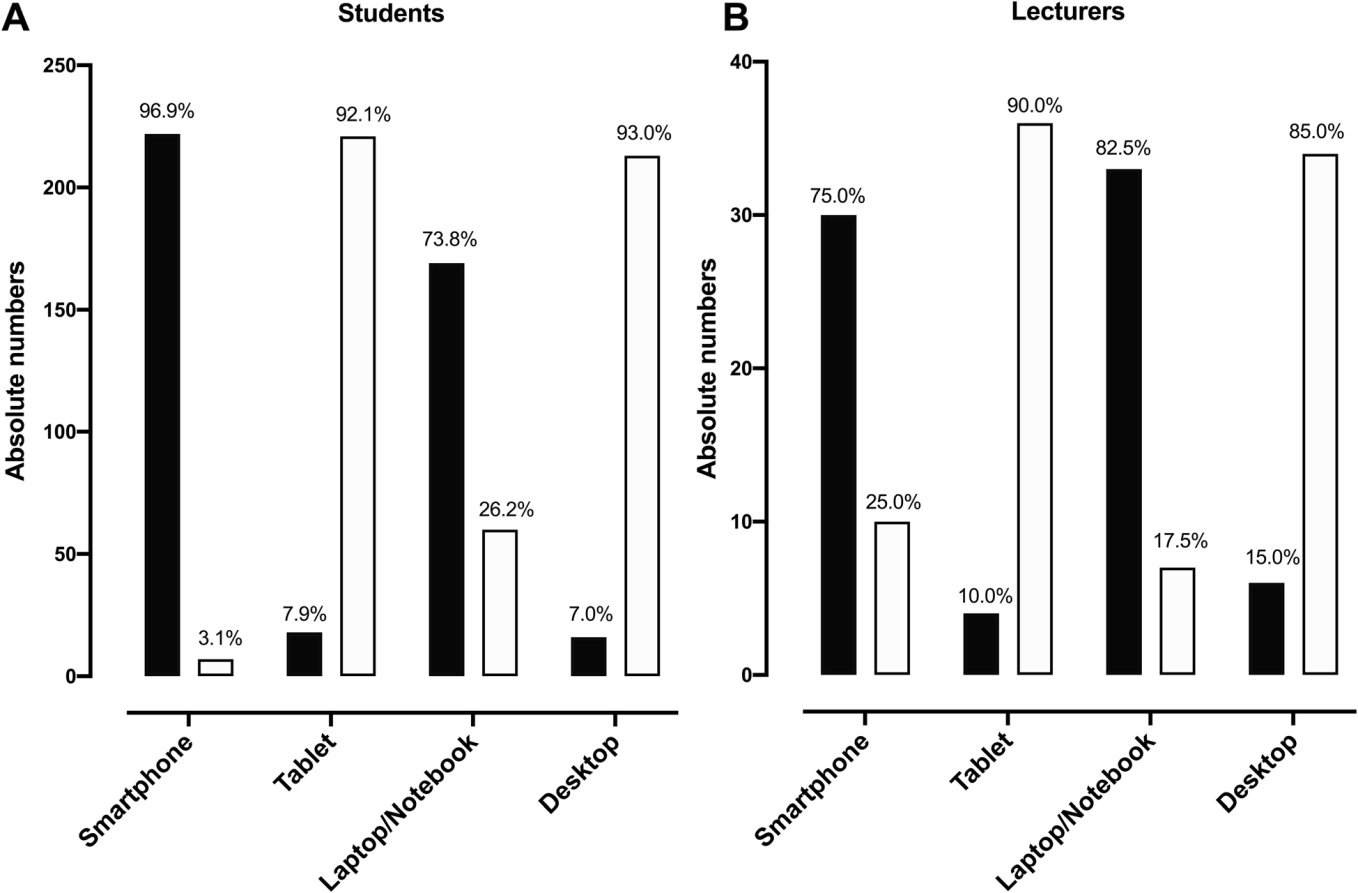
Electronic devices used for lectures by students (panel **A**) and lecturers (panel **B**). Black and white bars represent the number of students or lecturers who possess (black bars) or not (white bars) the various devices, respectively. Percentages corresponding to the numbers on the y-axis are represented above the bars of each of the devices

At the time of the survey and implementation of online lessons using WhatsApp®, one lecturer and seven students did not have a smartphone. However, they had a laptop or a notebook to comply with teaching duties and to follow lectures respectively. Students and faculty members mainly used either a smartphone [n(%) = 222(96.9) and 30(75.0), respectively] or a laptop/notebook [n(%) = 169(73.8) and 33(82.5), respectively], Fig. 1 A and B.

**Fig. 2:**
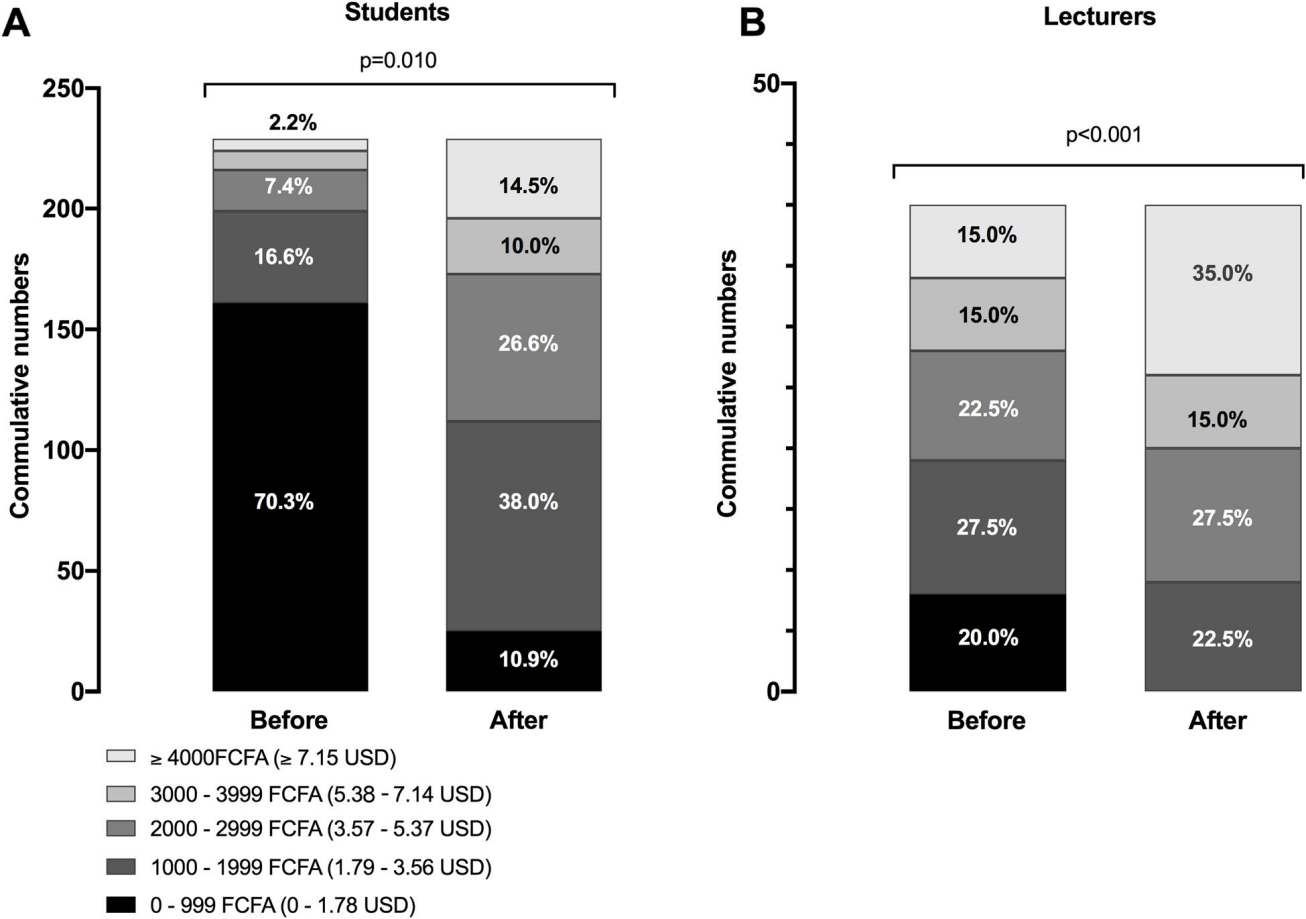
Monthly expenditure of internet connection in students’ (panel **A**) and lecturers’ (panel **B**) population before and after implementation of internet-based lectures using WhatsApp®. Cumulative numbers of participants are expressed in the y-axis for each study group. The proportion of individuals for each expenditure level are expressed as percentages

As expected, monthly expenditure related to internet use significantly increased both in the students’ population (*p* < 0.001, p-homogeneity < 0.001) and among faculty members (*p* = 0.010, p-homogeneity = 0.001). Baseline monthly expenses related to internet use was less than 1, 000 XAF (USD 1.79) for 70.3 % of students. During the implementation of online-lessons, 75.5 % of them spent up to 2, 999 XAF (USD 5.35) per month to access the internet, Fig. 2 A. On the other hand, the use of social media-based distance learning increased the expenses related to internet access to more than 4, 000 XAF (USD 7.15) in more than a third of lecturers, Fig. 2B.

**Fig. 3:**
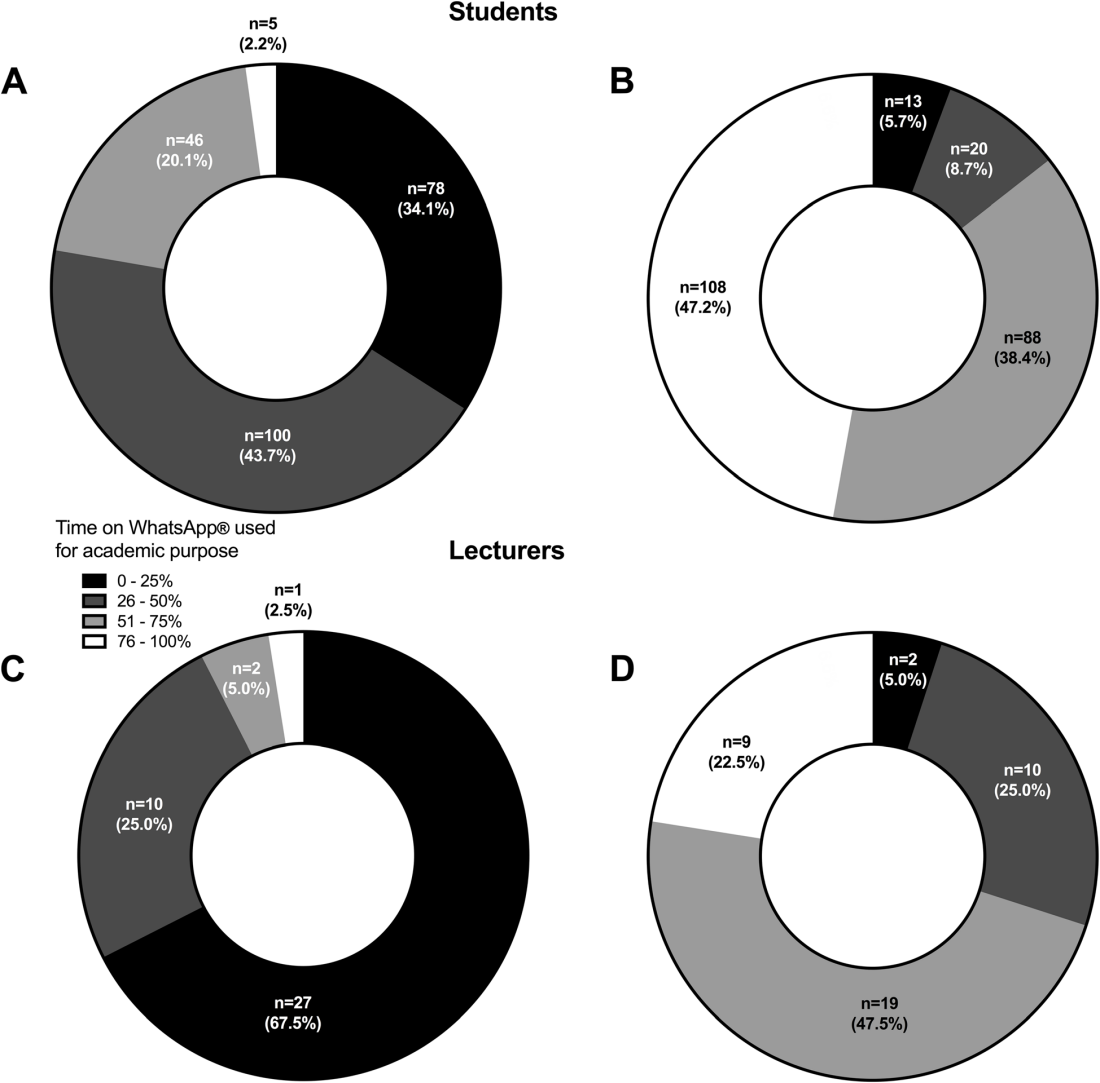
Proportion of time spent on WhatsApp® for academic purpose by students (upper panels) and lecturers (lower panels) before (panel **A** and **C**, respectively) and after (panel **B** and **D**, respectively) starting internet-based lectures

At baseline, 22.3 % (*n* = 51) of students used more than 50.0 % of their time on WhatsApp® for academic purposes. This proportion increased to 85.6 % (*n* = 196; overall *p* < 0.001, p-homogeneity < 0.001), Fig. 3 A and B during implementation of e-learning. The corresponding proportions in lecturers were lower than in the students’ group before [n(%) = 3 (7.5), *p* = 0.001, Fig. 3 C] and during e-learning period [n(%) = 28(70.0), *p* < 0.001, Fig. 3D]. Similar to students, the use of WhatsApp® for academic purpose increased among faculty members with the enforcement of distance learning at the FMPS of UDs (*p *< 0.001, p-homogeneity < 0.001), Fig. 3 C and D.

### Challenges and opinion of respondents about internet-based lectures

Despite a relatively good internet connection, students and lecturers faced difficulties either to download or upload lecture materials via the social media network. This challenge was more encountered among students and was mainly related to the files’ size and format, internet connection and cost (all p ≤ 0.015).

Opinions of both study groups were similar about discipline during lessons (all *p* > 0.05), Table 2. While lecturers’ feeling was that they were more comfortable answering questions during distance learning [n(%) = 16(40.0)], students felt less prone to ask questions [n(%) = 137(59.8)]. The motivation related to distance learning was at most similar to classroom-based lectures in the majority of students and lecturers. Moreover, students were globally less motivated by distance learning compared to lecturers [n(%) = 183(79.9) vs. n(%) = 14 (35), *p* < 0.001]. The observation was similar when satisfaction was explored [n(%) = 191(83.4) vs. n(%) = 25(62.5), *p* < 0.001]. The majority of students [n(%) = 191(83.4)] and lecturers [n(%) = 25(62.5)] were less satisfied by distance learning compared to traditional classroom-based lectures with a significant proportion among students (*p* = 0.002). However, most of the students [n(%) = 177(77.3)] and lecturers [n(%) = 37(92.5)], with a higher proportion of lecturers (*p* = 0.028), had a positive opinion about the practical organization of online lectures. Students [n(%) = 208(90.8)] and faculty members [n(%) = 26(65.0)] suggested in-person lectures to complement e-learning.

**Table.2:** Challenges and students’ opinion compared to that of lecturers about the performance and the organization of internet-based lectures using WhatsApp® during the COVID-19 pandemic

Characteristics	Students	Lecturers	P-value
Difficulties uploading or downloading files, n(%)	192 (83.8)	28 (70.0)	0.036
Files size, n(%)	159 (60.4	16 (42.1)	0.001
Format of files, n (%)	96 (41.9)	8 (21.0)	0.015
Connection, n (%)	201 (87.8)	20 (51.3)	< 0.001
Cost of connection, n (%)	188 (82.1)	14 (36.8)	< 0.001
Sight difficulties, n (%)	18 (7.8)	36 (15.4)	0.128
***Conduct of lectures***			
Opinion about students’ discipline compared to classroom-based learning			
Less discipline, n (%)	96 (41.9)	15 (37.5)	0.600
Similar discipline, n (%)	91 (39.7)	21 (52.5)	0.131
More discipline, n (%)	42 (18.3)	4 (10)	0.196
Opinion about students’ contribution compared to classroom-based learning			
Less contribution, n (%)	109 (47.6)	26 (65.0)	0.042
Similar contribution, n (%)	49 (21.4)	9 (22.5)	0.876
More contribution, n (%)	71 (31.0)	5 (12.5)	0.016
Opinion about lesson’s inputs to improve comprehension* compared to classroom-based learning			
Less, n (%)	137 (59.8)	9 (22.5)	-
Similar, n (%)	85 (37.1)	15 (37.5)	-
Better, n (%)	7 (3.1)	16 (40.0)	-
Motivation compared to classroom-based lectures			
Less motivation, n(%)	183 (79.9)	14 (35.0)	< 0.001
Similar motivation, n(%)	39 (17.0)	23 (57.5)	< 0.001
More motivation, n(%)	7 (3.1)	3 (7.5)	0.171
Satisfaction compared to classroom-based lectures			
Less satisfaction, n(%)	191 (83.4)	25 (62.5)	0.002
Similar satisfaction, n(%)	28 (12.2)	11 (27.5)	0.011
More satisfaction, n(%)	10 (4.4)	4 (10)	0.139

## Discussion

In this report, we share our experience on the implementation of mobile-learning, the perception and difficulties encountered by students and lecturers at the FMPS. Mobile-learning was used as a remediation strategy to cover the academic program during university lockdown due to the COVID-19 pandemic in Cameroon. Our results suggest that teaching equipment was largely available among students and faculty members. The major challenges related to the implementation of distance learning using WhatsApp® in our study population were to access internet and to share teaching materials. Despite a pretty good satisfaction about the organization, this teaching alternative warrants complementary measures to be as attractive as conventional classroom-based learning method for students in our study population.

Smartphone ownership rates were high among students and teachers. Several studies have found a high availability and use of smartphones among medical students in many countries [7]. This was an important prerequisite for the implementation of mobile-learning. Almost three out of four students spent at least four hours a day on WhatsApp® while this proportion corresponded to 43.6 % and 51.9 % of students at Albaha and Dammam universities, Saudi Arabia, respectively [8]. This extended time on WhatsApp® is not necessarily linked to the implementation of mobile-learning since students use this application also for the purpose of social exchanges. As discussed, by Alkhalaf MA et al., this can be a source of addiction, sleep disorders and poor academic performance [8, 9]. Therefore, in the case of concomitant use for social and academic purposes, students should be reminded of the risks associated with prolonged use of this application.

In a resource-limited setting such as that of our study, one needs to consider the increase in the internet connection budget. This could be a limiting factor for the participation of students in online teaching. However, the expenditure must be weighed against the reduction of other costs, those related to transport for example, which could have been reduced due to the lockdown. That notwithstanding, the financial difficulties are not be underestimated in the implementation of online lectures. Indeed, a similar study to ours from Libya, in the midst of the pandemic, conducted in 13 universities medical schools reported financial difficulties in about 40.5 % of students [10]. The various reason of this could be related to the gross domestic product per habitant of the country defining population’s income or to geopolitical conditions such as in Libya [10]. Besides its cost, the quality of internet connection before and during the lockdown is an additional important factor to consider. A slow Internet connection wastes time, increases the cost of connection and can be a source of poor scoring of e-learning for both students and teachers. Indeed, one of our observations is the challenge of downloading and uploading course materials. Although both students and lecturers increased their expenses related to internet use, this did not positively impact the quality of the connection. Considering the difficulties encountered by students and teachers in managing online courses, the major challenges were linked to the quality and cost of the internet connection. Among students, internet speed and related expenses were a challenge for 87.8 % and 82.1 %, respectively. These difficulties were significantly greater for students than lecturers (both *p* < 0.001). In a Liberian study about introducing e-learning in medical education, the authors found that limited bandwidth was one of the main limitations of distance learning [11]. Similarly, another report from Jordan indicated that internet streaming quality was the main challenge in the implementation of online lectures during the COVID-19 pandemic in 69.1 % of students [12].

Among students, 96 (41.9 %) felt that there was less discipline during online lessons than during face-to-face lectures. The difference in the perception of discipline from the perspective of students and that of teachers 21 (52.5 %) was not significant. While it is true that online teaching gives an impression of freedom (since it can be followed from home), participants should be self-disciplined and teachers should regularly remind students the rules applicable to this specific teaching method. Indiscipline here can take more subtle forms such as not logging in at the right time or logging out during class without a grounded reason [13]. In our study population, students and lecturers were rather satisfied with the modalities and organization of e-learning at the FMPS during the lockdown. However, the majority of surveyed students declared to be less satisfied, less motivated and less prone to actively participate in lectures compared to traditional classroom-based lessons. This could be related to the field and level of study of students included in the present survey. Moreover, the intrinsic nature of e-learning which closely relates to students’ affect cannot be underestimated [14, 15]. In fact, the vast majority of included students were undergraduate students. One could expect less propension to independent work, more need of contact with the tutors and peer interaction in this population [16]. Our data also suggest that 87.0 % of our study population thought that add-on classroom-lectures would improve e-learning using WhatsApp®. Although consistent with previous reports, this needs to be explored in subsequent studies considering as endpoints students’ and lecturers’ satisfaction and the impact on students’ performance [17].

Our survey has limitations that need to be acknowledged. The study was conducted in one-third of the overall students’ population from different fields and years of studies at the FMPS. This could suggest a selection bias related to the relatively low response rate and the heterogeneity of our study population. However, the absolute number of responses should be acknowledged. Furthermore, there is no formal consensual threshold sample size to be acceptable in such studies [18]. Also, postgraduate students are underrepresented in our study population. In this subset of students, one would expect more autonomy to appraise and to follow online lessons. Moreover, one could have considered assessing other determinants of students’ satisfaction, discipline and contribution during lessons which represent a potential source of bias in our data. For this, a larger study will be required in the future to refine the results and provide additional data. The strengths of our study include the high response rate of lecturers (100 %) and the survey of a heterogeneous group of students considering their years and fields of study. Nevertheless, the impact of level and type of study on the need of complementary lectures using conventional learning method needs to be explored in subsequent studies. Besides, the short term implementation of questionnaire after the conduct of online lectures has enabled us to avoid recall bias and has increased the accuracy of the data collected.

## Conclusions

In the context of the COVID-19, social media-based e-learning could be an applicable alternative to conventional classroom-based lessons. However, solutions to improve students’ satisfaction and motivation need to be sought. This could potentially be addressed by defining a blended-learning strategy where a specific number of hours of classroom-based courses could complement online lesson,. The effectiveness of this strategy to optimize delivery and assimilation of academic programs and to impact academic performances needs to be investigated.

## Supplementary information

Additional file 1 Supplementary material 1

Additional file 2 Supplementary material 2

## Data Availability

The datasets used and/or analyzed during the current study are available from the corresponding author on reasonable request.
