# Supplementary material for: Use of WhatsApp®, for distance teaching during COVID-19 pandemic: Experience and perception from a sub-Saharan African setting
Source: BMC Med Educ. 2021 Oct 2;21:517. doi: 10.1186/s12909-021-02953-9 (PMC8486629; doi:10.1186/s12909-021-02953-9)
Supplement: Supplementary file 2 — Supplementary material 2 [file 12909_2021_2953_MOESM2_ESM.doc]

Use of WhatsApp®, a simple and widely distributed social media, for distance

teaching during COVID-19 pandemic: Experience and perception from a sub-

Saharan African setting

Use of WhatsApp®, a simple and widely distributed social media, for distance

teaching during COVID-19 pandemic: Experience and perception from a sub-

Saharan African setting

**Supplemental material 2**

**Use of WhatsApp®, a simple and widely distributed social media, for distance teaching during COVID-19 pandemic: Experience and perception from a sub-Saharan African setting**

Dominique Enyama ^1, 2, 8^, Eric Vounsia Balti ^1, 3, 8^, Sylvain Raoul Simeni Njonnou ^1, 8^, Christian Ngongang Ouankou ^1, 4^, ^8^, Fernando Kemta Lekpa ^1, 5, 8^, Diomede Noukeu Njinkui ^1, 2, 8^, Jovanny Tsuala Fouogue ^1, 6, 8^ Jeanne Mayouego Kouam^1^, Guy Sedar Singor Njateng ^7, 8^, Bruno Kenfack ^1, 8^, Pierre Watcho ^8^, Simeon Pierre Choukem ^1, 8, 9^

^1^ Department of Clinical Sciences, Faculty of Medicine and Pharmaceutical Sciences, University of Dschang, Dschang, Cameroon

^2^ Department of Pediatrics, Douala Gyneaco-Obstetric and Pediatric Hospital, Douala, Cameroon

^3^ Department of Internal Medicine, Universiteit Ziekenhuis Brussel, Vrije Universiteit Brussel, Brussels, Belgium

^4^ Yaoundé University Teaching Hospital, Yaoundé, Cameroon

^5^ Department of Internal Medicine, Douala General Hospital, Douala, Cameroon

^6^ Department of Obstetrics and Gynaecology, Bafoussam Regional Hospital, Cameroon

^7^ Department of Biochemistry, Faculty of Sciences, University of Dschang, Dschang, Cameroon

^8^ The University of Dschang Taskforce for the Elimination of COVID-19 (UNITED#COVID-19), Dschang, Cameroon

^9^ Health and Human Development (2HD) Research Network, Douala, Cameroon

*Contact author:*

Prof. Simeon Pierre Choukem

Department of Clinical Sciences, Faculty of Medicine and Pharmaceutical Sciences, University of Dschang, Dschang, Cameroon

E-mail: [simeon.choukem@univ-dschang.org](mailto:simeon.choukem@univ-dschang.org)

*Keywords:* WhatsApp®, social media, e-learning, online-learning, distance learning, higher education, perception

**Lecturers’ survey form**

I – Socio-demographic data

1. You are: *

*Choose only one answer*


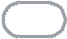
 A man


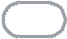
 A woman

1. The year you were born is:
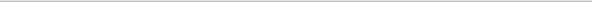
*
2. What is your department: *

*Choose only one answer*


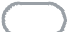
 Public health


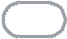
 Pharmaceutical Sciences


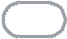
 Microbiology, hematology and immunology


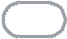
 Surgery and specialties, anaesthetics and critical care


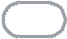
 Physiology and biochemistry


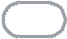
 Internal Medicine and specialties


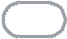
 Paediatrics


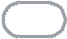
 Gynaecology and obstetrics


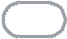
 Ophthalmology, KNO and stomatology


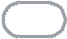
 Radiology


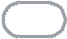
 Physiotherapy

1. Do you have another job? * *Choose only one answer*


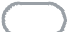
 Yes


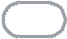
 No

1. If yes, indicate in which type of facility*

*Choose all what apply*


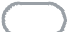
 Hospital


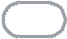
 Private clinic


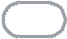
 Laboratory


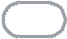
 Administration


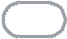
 Other

1. At the Faculty of Medicine and Pharmaceutical Sciences of University of Dschang in which group of lecturers are you ? *

*Choose only one answer*


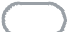
 Permanent


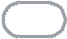
 Visiting

II – Common use of electronic devices

1. Do you have: *

*
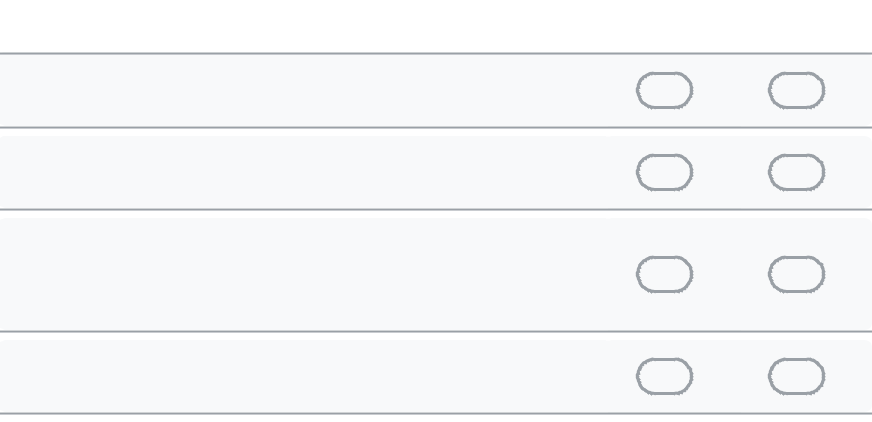
*

Yes No

**A Smartphone**

**A Tablet**

**A Portable Computer (**L**aptop or**

**Notebook)**

**A Desktop**

1. Had you already installed WhatsApp® application on your Smartphone or computer before the beginning of the online-teaching period at the Faculty of Medicine and Pharmaceutical Sciences? *

*Choose only one answer*


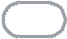
 Yes


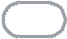
 No

1. How much time do you spend daily on WhatsApp®? *

*Choose only one answer*


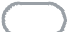
 < 2 hours/day


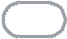
 2 – 4 hours/day


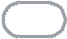
 4 – 6 hours/day


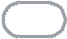
 6 – 8 hours/day


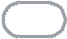
 8 – 10 hours/day


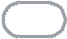
 10 – 12 hours/day


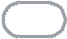
 >12 hours/day

1. BEFORE the beginning of the online-teaching period, for which purposes did you usually use WhatsApp®? *

*
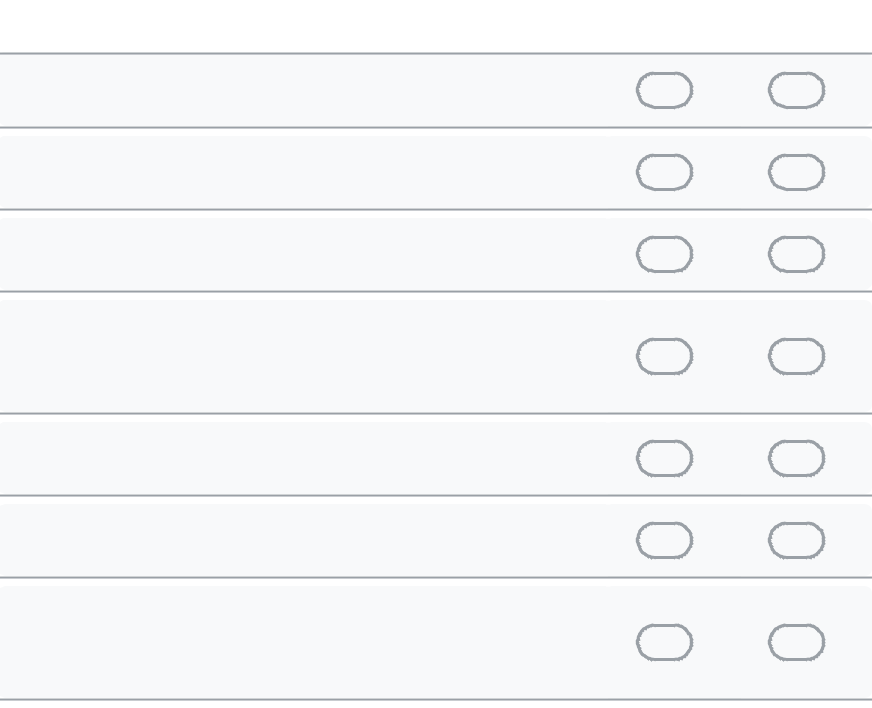
*

Yes No

**To send text messages**

**To send pictures**

**To send videos**

**To share news**

**To send voice messages**

**To prepare assessment**

**To discuss homework with classmates**

1. BEFORE the beginning of online-teaching period, how did you use WhatsApp® for academic purposes (Estimate the average time spent on internet)? *

*Choose only one answer.*


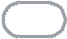
 0 -25%


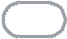
 26 - 50%


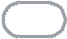
 51-75%


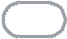
76 -10%

1. BEFORE the beginning of the online-teaching period, how much did you spend each week for internet? *

*Choose only one answer.*


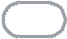
 0 – 999 francs CFA (0 – 1.78 USD)


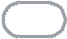
 1000 – 1999 francs CFA (1.79 – 3.56 USD)


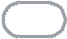
 2000 – 2999 francs CFA (3.57 – 5.37 USD)


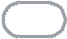
 3000 -3999 francs CFA (5.38 - 7.14 USD)


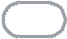
> 4000 francs CFA (≥ 7.15 USD)

1. SINCE the beginning of the online-teaching period, how do you use WhatsApp® for academic purposes (Estimate the average time spent on internet)? *

*Choose only one answer.*


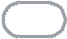
 0 -25%


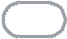
 26 - 50%


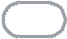
 51 - 75%


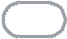
76 - 100%

1. SINCE the beginning of the online-teaching period, how much do you spend each week for internet connection? *

*Choose only one answer.*


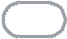
 0 – 999 francs CFA (0 – 1.78 USD)


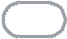
 1000 – 1999 francs CFA (1.79 – 3.56 USD)


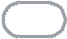
 2000 – 2999 francs CFA (3.57 – 5.37 USD)


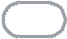
 3000 -3999 francs CFA (5.38 - 7.14 USD)


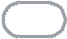
> 4000 francs CFA (≥ 7.15 USD)

1. How would you describe the flow of your internet connection BEFORE the lockdown? *

*Choose only one answer.*


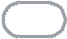
 Very good


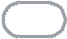
 Good


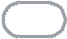
 Fair


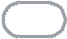
 Poor

1. How would you describe the flow of your internet connection SINCE the lockdown? *

*Choose only one answer.*


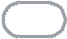
 Very good


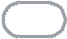
 Good


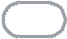
 Fair


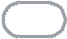
 Poor

III – Online-teaching

1. Which electronic devices do you use to take courses during the online-teaching period? *

*
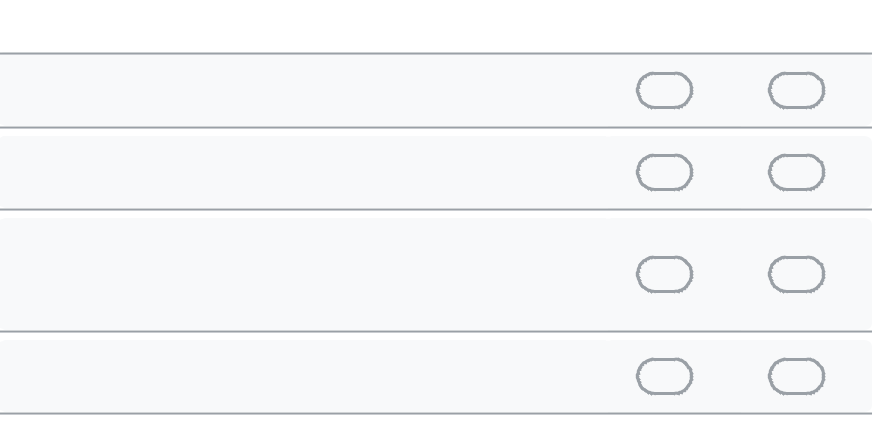
*

Yes No

**A Smartphone**

**A Tab**l**et**

**A portable computer (**L**aptop or Notebook)**

**A Desktop**

1. Concerning the course materials provided during the online-teaching period, which file format looks more appropriate to you? *

*
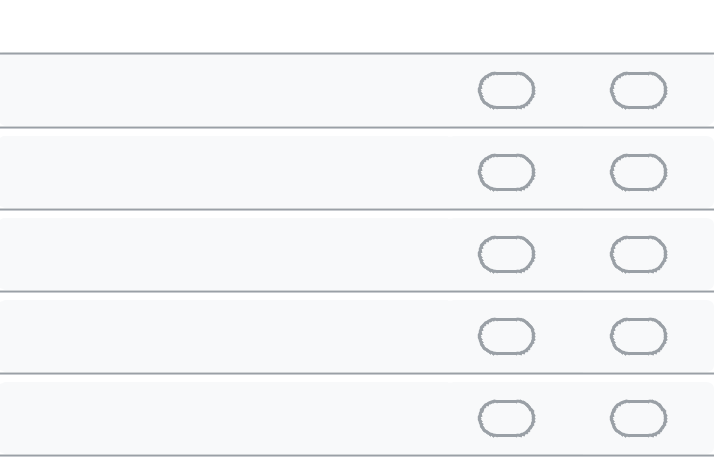
*

Yes No

**PDF**

**Word**

**PowerPo**i**nt**

**V**i**deo**

**Aud**i**o**

1. Among the following communication channels, which one was most used for discussions during the courses? *

*Choose only one answer.*


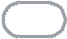
 Text messages


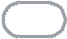
 Voice messages


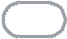
 Video messages


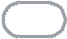
 Other :


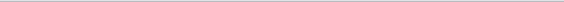


1. Among the following communication channels, which one looks more appropriate to you for discussions during the courses? *

*Choose only one answer.*


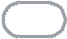
 Text messages


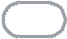
 Voice messages


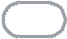
 Video messages


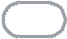
 Other

1. Regarding lectures planning during the online-teaching period, which option was more preferable for you? *

*Choose only one answer.*


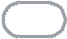
 Keep the same time quotas as for face-to-face courses


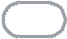
 Plan less time


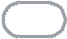
 Plan more time

1. When is it preferable to provide the course materials for the scheduled lectures? *

*Choose only one answer.*


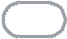
 One week before the scheduled date of the course


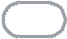
 The day before the scheduled date of the course


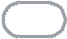
 Some hours before the scheduled date of the course

1. Do you think that ALL the courses should be given through online-teaching via WhatsApp®? *

*Choose only one answer.*


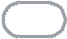
 Yes


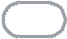
 No

1. Do you find it pertinent to create a WhatsApp® group for each topic or lecture? *

*Choose only one answer.*


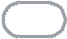
 Yes


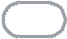
 No

1. Do you think that WhatsApp® groups could be used to continue discussions on topics covered during face-to-face courses? *

*Choose only one answer.*


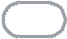
 Yes


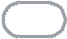
 No

1. What is your general appreciation of the QUALITY of lectures given via WhatsApp® during the online-teaching period? *

*Choose only one answer.*


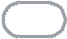
 Very poor


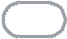
 Poor


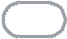
 Fair


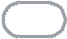
 Good


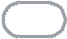
 Very good

1. What is your general appreciation of the ORGANISATION of lectures given via WhatsApp® during the online-teaching period? *

*Choose only one answer.*


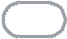
 Very poor


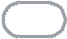
 Poor


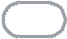
 Fair


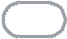
 Good


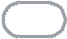
 Very good

1. In general, are you satisfied with the use of WhatsApp® for online-teaching during the lockdown? *

*Choose only one answer.*


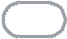
 Not satisfied


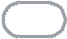
 Satisfied


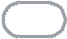
 Very satisfied

1. What would you prefer between teaching via WhatsApp® and face-to-face teaching? *

*Choose only one answer.*


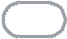
 Teaching via WhatsApp® is preferable


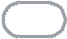
 Face-to-face teaching is preferable


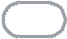
 No preferance

1. What proportion of the total number of WhatsApp® based courses have you taken? *

*Choose only one answer.*


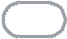
 0-25%

26 - 50%

51- 75%

76 - 100%

1. Do you think that online courses are sufficient to assess students? *

*Choose only one answer.*

Yes

No

1. If no, why ?
2. If not, what can be done? *

*Choose only one answer.*

Repeat each lesson during face-to-face courses

Plan face-to-face revision

1. If you have chosen face-to-face revision, how much time should be allocated to revisions compared to the duration of the course given? *

*Choose only one answer.*

1 hour of face-to-face revision for 2 hours of online course

1 hour of face-to-face revision for 4 hours of online course

1 hour of face-to-face revision for 6 hours of online course

1 hour of face-to-face revision for 8 hours of online course

Other

1. If other, specify

IV – Difficulties encountered

1. Have you faced any difficulties to upload course materials? *

*Choose only one answer.*

Yes

No

1. Which difficulties did you encountered among the following? *

Yes No

**Files size**

**Files fomat**

**Poor internet connection**

**Cost of internet connection**

**Lack of Smartphone**

**Visual problems (Eyestrain due to screen exposure)**

1. Regarding discipline, how did you find the class during WhatsApp® courses? *

*Choose only one answer.*

More discipline compared to face-to-face class

Less discipline compared to face-to-face class

Same discipline compared to face-to-face class

1. Regarding participation, how did you find the class during WhatsApp® courses? *

*Choose only one answer.*

More participation compared to face-to-face class

Less participation compared to face-to-face class

Same participation compared to face-to-face class

V– Discipline, attendance and motivation

1. On face-to-face courses, In the allocated time, I usually cover about: *

*Choose only one answer.*

0 - 25% of the courses

26 - 50% of the courses

51 - 75% of the courses

76 - 100% of the courses

1. On WhatsApp® courses, In the allocated time, I usually cover about: *

*Choose only one answer.*

0 - 25% of the courses

26 - 50% of the courses

51 - 75% of the courses

76 - 100% of the courses

1. During WhatsApp® courses, I am usually online and available for student on average during: *

*Choose only one answer.*

0 - 25% of the course duration

26 - 50% of the course duration

51 - 75% of the course duration

76 - 100% of the course duration

1. Do you feel more comfortable answering questions from students on WhatsApp® compared to face-to-face courses? *

*Choose only one answer.*

Yes

No

Similar

1. How did you find the teaching via WhatsApp®: *

*Choose only one answer.*

More motivating than face-to-face courses

Less motivating than face-to-face courses

Same motivation regardless the mode of teaching

1. What is your overall satisfaction with the courses via WhatsApp® compared to face-to-face courses? *

*Choose only one answer.*

Less good

Similar

Better
